# Use the Environment to Prevent and Control COVID-19 in Senior-Living Facilities: An Analysis of the Guidelines Used in China

**DOI:** 10.1177/1937586720953519

**Published:** 2020-09-10

**Authors:** Zhe Wang

**Affiliations:** 1 Department of Architecture, 12411 Henan University, Kaifeng, China

**Keywords:** older adults, environmental design, infection control, senior living, long-term care facilities

## Abstract

**Objective:**

To identify the environmental factors essential for infection control in senior-living facilities.

**Background:**

In the COVID-19 pandemic, older adults are more likely to be infected and develop serious outcomes than young people. Worldwide, senior-living facilities face a battle to protect their residents. Compared with age-related declines, the built environment is more modifiable and can be used for infection control.

**Methods:**

This research conducted content analysis of the guidelines on COVID-19 control issued by the State Council of China in February 2020 for senior-living facilities. Six senior-living facility managers in China were interviewed and shared their experiences using these guidelines. Quantitative and qualitative analyses were conducted to identify the essential environmental factors for infection control.

**Results:**

Environmental factors suggested in the guidelines were analyzed for three groups of infection-control strategies: keep COVID-19 from entering the facility, prevent COVID-19 spread in the facility, and manage infection and illness. Key topics of experience using the guidelines were identified, including residents’ needs for social interaction and the difficulties of providing dedicated air-conditioning and circulation systems. Based on these analyses, from the perspective of environmental design, environmental factors essential for COVID-19 control in senior-living facilities were summarized at the site, building, and room levels.

**Conclusion:**

Proper planning and design of the built environment promote strategies for infection control in senior-living facilities. Findings can be used to guide the new design, renovation, and modification of senior-living facilities for COVID-19 control and future public health emergencies.

By August 2020, the number of confirmed cases of coronavirus disease (COVID-19) has reached 23.9 million worldwide (Johns Hopkins University, 2020). Older adults and people with underlying health conditions are more likely to be infected and develop serious outcomes versus otherwise younger and healthier people (McMichael et al., 2020). As aging is a global condition, all countries take proactive steps to protect older adults.

The largest populations of people aged 80+ (also called the oldest old) live in China (23 million) and the United States (12 million) (United Nations, 2015). In March 2020, the United States passed China to become the country with the most confirmed cases of COVID-19 in the world (JHU, 2020). There are 8.4 million Americans living in long-term care facilities; 85% of them are older adults, and 42% aged 80+ (Harris-Kojetin et al., 2016). Over two thirds of Americans who reach age 65 will need long-term care services during their lifetime (Kemper et al., 2006). According to recent reports, of all deaths reported in the United States, more than 40% have been linked to coronavirus outbreaks in long-term care facilities (The Society for Post-Acute and Long-Term Care Medicine, 2020; Condon & Herschaft, 2020). In China, the number of senior-living beds has increased from 3.7 million to 7.8 million in the recent 5 years (Ministry of Civil Affairs of China, 2020; National Bureau of Statistics of China, 2015). The fatality rate of COVID-19 infections in Chinese aged 80+ was 21.9%, whereas the overall was 3.8% of all ages nationwide (Kupferschmidt & Cohen, 2020; Wu, 2020). Cross-nationally, senior-living facilities face an uphill battle to keep their residents from this virus, serious illness, complications, and death.

There are a variety of contributors to this problem and some of which are hard to change, such as age-related functional declines and chronic diseases. Older adults’ immune system gradually deteriorates as age increases, making it harder for the body to fight off diseases and infection (Morley & Vellas, 2020). Many older adults have underlying conditions that hinder their ability to cope and recover from illness. Some also experience cognitive declines and communication difficulties, leading to delays in medical treatment. In elderly patients, antibiotics are less effective, and the rate of cure after infection is lower than in other age groups (Loeb et al., 2002). Moreover, the risk of infection through person-to-person contact is high in older adults since close physical contact between older adults and their caregivers is typically required. This also reduces the possibilities to keep older adults in quarantine by themselves. Associated with these factors, infection control in senior-living facilities isn’t easy. In Washington State, 650 nursing homes reported having residents with COVID-19 and 40% of them have already been cited for infection-control infractions before the reports (Cenziper et al., 2020; Ingold, 2020).

Compared with age-related declines, the built environment is more modifiable and should be used to support infection control. The strategies for infection control can be categorized into personal, social, and environmental strategies (Altman & Wohlwill, 2012; Bonell et al., 2020). From the perspective of environment and behavior, personal and social behaviors for infection control should be supported by the environment. Compared with emergency management strategies, environmental strategies for infection control can be more sustainable and long lasting. It is of urgent need to identify essential environmental factors for COVID-19 control in senior-living facilities. Given the confirmed drops in COVID-19 spread in China (Kupferschmidt & Cohen, 2020), it is worth to analyze the infection-control strategies applied in China. This research analyzed the guidelines on COVID-19 control issued by the State Council of China in February 2020 for senior-living facilities. Six senior-living facility managers in China were interviewed and shared their frontline experiences using these guidelines. Based on data analyses, from the perspective of environmental design, environmental factors essential for COVID-19 control in senior-living facilities were summarized at the site, building, and room levels.

***Compared with age-related declines, the built environment is more modifiable and should be used to support infection control***.

## Guidelines Applied in China

In China, many senior-living facilities went into lockdown in late January 2020. On February 7th and 25th, the State Council and the Ministry of Civil Affairs issued the second edition of “Guidelines for Preventing and Controlling COVID-19 in Senior-Living Facilities” and the “Guidelines for Preventing and Controlling COVID-19 in Infected Senior-Living Facilities and High-Risk Areas” (China, 2020a, 2020b). Compared with the previously applied guidelines, the new guidelines are more detailed, stricter on facility management, and require more protection. From facility leadership to specific measures for disinfection, there are 35 specific points on COVID-19 prevention and control. Directed by local governments, most senior-living facilities in China have used these guidelines. Following the drops in COVID-19 spread, many facilities in low-risk areas started ending the lockdown in late March.

According to the behavior setting theory, needs for appropriate environments can be viewed as the interactions between people and their environments along spatial and temporal dimensions (Barker, 1968). The interactions in a senior-living facility determine its ability to prevent and control COVID-19. Using this theory as a conceptual framework, this research conducted content analysis on these guidelines and categorized the suggested infection-control and prevention strategies into three groups: keep COVID-19 from entering the facility, prevent COVID-19 spread in the facility, and manage infection and illness.

***…keep COVID-19 from entering the facility, prevent COVID-19 spread in the facility, and manage infection and illness***.

### Keep COVID-19 From Entering the Facility

To keep COVID-19 from entering the facilities, the guidelines suggested strict restrictions on entering. No vehicles are allowed to visit a senior-living facility, except those for special reasons (e.g., end of life situations). Vehicles must be disinfected before entering and park at designated locations in the facility. Outside the facility, a loading zone or area should be established for material handover (e.g., supplies from family members, items ordered online). Delivery personnel should wear appropriate personal protective equipment (PPE), pass a temperature test for fever, and conduct hand hygiene, sole disinfection, and other protective measures. Protected by appropriate PPE, staff members working in the facility shall spray chlorine-containing disinfection liquid or 75% medical ethanol (for small items) on the materials intended to enter the facility. No visitors should be allowed, except those for special reasons (e.g., compassionate care). All service activities requiring facility visiting should be suspended. If allowed and before entering, visitors should register, be screened for fever and respiratory symptoms, and confirm no travel to high-risk areas in the past 14 days. Visitors should wear appropriate PPE, conduct hand hygiene, stay in designated areas such as a reception room, and comply with other prevention and control requirements asked by the facility.

During the time of facility lockdown, staff members should live inside the facility to avoid reentering. If needed, staff members may stay in a dedicated area outside the facility, which should be separated from people living outside the facility. Single rooms were suggested for staff members to reduce the risk of infection. Those with suspected symptoms shall be sent to a designated hospital for medical treatment. Dedicated shuttles should be provided for staff’s commute, and no public transportation should be used. No new staff members or healthcare personnel should be hired, except for special reasons. The newly hired or existing members returning from hospital stay should complete a 14-day quarantine and have nucleic acid testing normal before entering the facility. Senior-living residents were asked to cancel their travel plans. No new residents should be admitted, except those for special reasons (e.g., with dementia and loss of all family support). The newly admitted or those who traveled should complete a 14-day quarantine and have nucleic acid testing normal before entering the facility.

Environmental factors relevant to the aforementioned strategies include loading zones or areas for material handover outside the facility, designated parking areas for visiting vehicles, registration spaces for visitor screening, designated areas for visitors’ stay, and dedicated areas and single rooms for staff’s facility stay.

### Prevent COVID-19 Spread in the Facility

Environments in a senior-living facility should be categorized into contaminated, semi-clean, and clean zones for infection-control purposes. The methods of zoning should be based on the current COVID-19 situation and behavior maps of residents, staff members, and visitors. These zones should be physically separated by engineering controls. A dedicated air-conditioning system should be set up for each zone. In case a central air-conditioning system was used, the provisions of system disinfection required for medical protection (e.g., high-efficiency particulate air filters and ozone-treated heating, ventilation, and air-conditioning) should be strictly followed. Dedicated circulation systems including corridors and exits/entries should be set up for each zone to reduce unnecessary interactions and enhance zoning management. Zoning policies and operation procedures should be widely introduced and clear signage on walls designating different zones may be used.

For natural ventilation, window(s) in a residential room should be open for at least 30 min per 12 hr. Attention should be paid to avoid catching colds caused by excessive temperature differences between the interior and outdoors. If needed, mechanical ventilation (e.g., using a fan) directly with the outdoor air should be conducted for air exchange at the room level. Room air disinfection using circulating air disinfection machines should be carried out if necessary, and visual room inspection should be conducted daily. Residents’ meals should be provided by a built-in kitchen in the facility or a designated canteen. If possible, group meals should be suspended, and all meals should be sent to residents’ rooms. Otherwise, separated small-group meals should be scheduled, and social distancing policies should be applied in dining rooms to avoid the spread of droplets. All surfaces including tables, chairs, door handles, faucets, switch buttons, handrails should be wiped with clean water once per day and disinfected using alcohol-based measures 1–2 times per week.

All gathering or group activities should be suspended. Visiting others’ rooms may not be allowed. Instead, internet access, television, radio, reading materials, and other cultural and entertainment services should be provided to residents in their rooms. Tele-interactions and communications (e.g., video calls) should be encouraged to relieve anxiety and guide residents to maintain their social networks and regular life. Residents in quarantine should be given priority in receiving psychological supports. Outdoor activities on facility’s site should be encouraged to promote active living.

Environmental factors relevant to the aforementioned behaviors include environmental zoning, dedicated circulation systems, natural ventilation, equipment for mechanical ventilation, built-in kitchens, furnishing for social distancing, outdoor environments for physical activities, and access to the internet for tele-interaction.

### Manage Infection and Illness

Senior-living facilities may not be adequately equipped to manage cases of COVID-19. Once any suspected case occurs, it is crucial to transfer the patient to a specialized hospital as soon as possible. Dedicated rooms should be provided for quarantine in the facilities before sending patients to hospital, such as during the time waiting for hospital beds to be available. Each patient should stay in a private room with good natural ventilation and separated from other residents’ rooms. Supplies for the patients, staff members, and healthcare personnel (e.g., physicians and nurses) should be configured according to the requirements for medical protection (e.g., protective gear, medical masks, gloves, and hand hygiene using alcohol-based measures). These patient rooms need to be grouped in a dedicated area, which also includes necessary circulation systems and is an important part of the contaminated zone. The dedicated patient area can be a building or part of a building, such as a dedicated wing. Location of the selected building or building part should be downwind (in the same direction toward which prevailing winds blow) from other buildings on the facility’s site or other spaces in the same building. Household waste in patient areas should be disinfected and separated from those in other areas.

If no urgent care is needed, telemedicine or doctor’s home visits should be used instead of sending residents outside the facility for medical treatment. In case of urgent needs, the patient shall be accompanied by family members or staff members under effective COVID-19 protection to a designated hospital. Residents’ needs for medical treatment should be handled individually and consulted with their family members.

Environmental factors relevant to the aforementioned behaviors include the following: dedicated private rooms for patients (relatively independent, well-ventilated, and located downwind on facility’s site), dedicated air-conditioning systems, dedicated areas for household waste, and access to telemedicine.

## Experience Using the Guidelines

To collect experience using the guidelines, the team conducted phone interviews with senior-living facility managers in China. Participant managers shared their experience using the guidelines and highlighted key topics that need special attention. This research was approved by the institutional review board at a university in China.

### Participant Facilities

Based on previously developed research networks, through phone call, WeChat (a mobile phone app), and email, the team sent interview invitations to 11 senior-living managers or directors in China. Six of them accepted the invitation and the rest had booked other activities during the time of the proposed interviews. Average age of these participants was in their 40s and four were female. Their education was at college level (on average). By the time of interview (May 2020), they had an average of 11 years’ experience in the field of senior-living services. Their facilities were located in Shanghai (city population of 22 million, in the eastern part of China), Zhengzhou (city population of 4.3 million, central part), and Kaifeng (city population of 0.9 million, central part) (Population Stat, 2019). These facilities had run for an average of 13 years and provided multiple levels of care including independent living, assisted living, and nursing care on one site. Their capacities ranged from 100 to 360 beds and the average occupancy rate was 79%, representing the major categories of senior-living facilities in China (Table 1).

**Table 1.** Participant Facilities During the Lockdown.

Facility	Year Built	Capacity	Existing Residents	COVID-19 Patients	In-House Healthcare Personnel	Guideline Use	Single Rooms for Patients	Single Rooms for Staff	Dedicated AC Systems	Dedicated Patient Areas	Dedicated Circulation Systems
1	2006	250	163	5	3	Yes	Yes	No	No AC	Yes	No
2	2009	360	272	8	8	Yes	Yes	No	Yes	No	No
3	1998	120	101	3	2	Yes	Yes	No	No	Yes	No
4	2013	100	89	3	2	Yes	Yes	No	No	Yes	No
5	2015	120	90	3	1	Yes	Yes	No	No AC	Yes	No
6	2006	150	131	2	1	Yes	Yes	No	No	Yes	No

*Note.* AC = air-conditioning.

### Interviews

Phone interviews were conducted by two researchers with a PhD or master’s degree majoring in architecture or environmental design and an average of 9 years’ experience in senior-living research. Average duration of these interviews was 21 min (range from 17 to 29 min). Participants shared their experiences using the guidelines in their facilities. Leading questions used in these interviews included: Did your facility use these guidelines? If so, what is your experience using these guidelines? Did you meet any challenges?

Participants’ facilities had used these guidelines since February 2020. At the time of interview, these guidelines were still applied and none of the facilities had COVID-19 outbreaks. The total number of suspected or confirmed cases ever reported in these facilities ranged from two to eight by facility. Directed by local government offices, these facilities ended the lockdown on April 7th or 25th and started allowing family members (no other visitors) to visit residents. Based on content analysis, key topics discussed by these participants were summarized: needs for social interaction, “in-house” medical services, challenges in providing dedicated air-conditioning and circulation systems, and spaces for staff’s facility stay.

### Key Topics

1. Needs of the residents and families for social interaction were highlighted by all participants. During facility lockdown, there were few group activities inside the facilities. Many residents were unhappy about it. Their families also contacted the managers and indicated their needs for visiting during the lockdown. To address the needs, facility managers created WeChat groups and held video conferences on varied online meeting platforms to facilitate tele-interactions between residents and their families. According to the participants, these tele-interactions were helpful, but more work should be done to address the needs.
2. Regarding medical services needed in their facilities during lockdown, five participants said some of the needs were not being met. They experienced lack of healthcare personnel and medical supplies including PPE. One manager said, “This epidemic tells us how important it is to have an ‘in-house’ medical team.” Associated with facility sizes and specific situations, the number of healthcare personnel varied from one to eight by facility. All of these facilities had joint service agreements with local hospitals. One facility worked on an “in-house” medical station that was expected to be built in 2020. It is a clinic built inside the facility, providing medical care services to both the residents and local communities, with an emphasis on gerontological care. In the relatively large facility with 272 residents, all residential rooms were private. The risk of person-to-person infection was thought to be low, and thus, a dedicated area for grouping patient rooms wasn’t set up in this facility. Instead, residents with suspected symptoms were asked to stay inside their existing rooms that were generally next to other residential rooms. By the time of interview, there were eight confirmed cases of COVID-19, and the rate of infection was relatively high in these participant facilities. More research is needed to investigate the possible reasons. The manager suggested that even if all residential rooms were private, a dedicated patient area for quarantine should be established.
3. Regarding air-conditioning, participants experienced challenges in providing dedicated air-conditioning systems by zone. Five of the six facilities were unable to do so since all of their rooms were designed to use a central air-conditioning system. The facility that provided dedicated air-conditioning systems used window air conditioners at the room level. Of the five, two stopped using air-conditioning systems since this January and used portable electric heaters to keep room temperature in the winter months. The other three worked hard to reduce the risk of infection by transferring patients to hospital in a short time. Regarding dedicated circulation systems suggested in the guidelines, none of the facilities could stop shared use of elevators or staircases, and thus, no dedicated circulation systems were provided. They followed the guidelines to clean and disinfect all surfaces in the circulation systems to reduce the risk of infection.
4. Dedicated staff areas and single rooms for staff’s facility stay were suggested in the guidelines. However, five facilities were lack of space to do so. They used staff offices to provide accommodation for their staff. These offices were designed to be associated with nurse stations for individual care groups and thus had close interaction with residents’ areas.

## Essential Environmental Factors for Infection Control

Based on the guidelines and managers’ empirical experiences using the guidelines, for each group of infection-control strategies, the essential environmental factors were summarized and presented by spatial level: site, building, and room (Table 2).

**Table 2.** Essential Environmental Factors for COVID-19 Control in Senior-Living Facilities.

Factors	Keep COVID-19 From Entering the Facility	Prevent COVID-19 Spread in the Facility	Manage Infection and Illness
Site-level	Loading zones/areas for material handover	Environmental zones (contaminated, semi-clean, and clean) for necessary separation	Built-in medical station
	Dedicated parking areas for visiting vehicles	Dedicated areas for staff’s facility stay	Access to telemedicine
	Signage systems for infection-control management	Dedicated areas for facility kitchen	Outdoor environments for physical activities
Building-level	Reception room for visitor screening and registration	Natural ventilation	
	Storage for devices needed for screening	Dedicated patient areas	
	Storage for disinfection supplies	Dedicated air-conditioning systems	
	Signage systems for infection-control management	Dedicated circulation systems	
		Storage for disinfection supplies and equipment	Designated space for household waste in patient areas
Room-level	Signage systems for infection-control management	Backup mechanical ventilation at the room level	
		Access to telemedicine, internet, television, radio, reading materials, and items for cultural and entertainment services	
		Storage for devices used in daily inspection	
		Dining room furnishing for social distancing	Relatively independent single rooms for patients

### At the Site Level

To prevent COVID-19 from entering the facility, a loading zone or area for material handover outside the facility was suggested in the guidelines, along with a dedicated parking lot inside the facility for visiting vehicles. To prevent and control infection, dedicated environmental zones including the clean (e.g., residential rooms), semi-clean (e.g., facility clinic), and contaminated (patient areas) zone should be considered at the level of site planning. Each zone should have independent air-conditioning systems, dedicated circulation systems (e.g., entries and exits), and routes for waste collection. Engineering separations between these zones would be necessary in the time of pandemic, and the spaces to store the materials for engineering separation should be included in planning.

Location of the contaminated zone should be downwind on the facility’s site and relatively separated from other buildings or spaces. A built-in medical station is necessary for both infection control and daily healthcare in senior-living facilities. Its location and specific functions should be carefully considered in the context of individual facilities. To facilitate telemedicine and tele-interaction, internet infrastructure should be considered at the level of site planning.

Dedicated areas for staff members’ facility stay during facility lockdown should be considered in planning. Multipurpose spaces in the interior or outdoors, such as park sites for portable temporary housing, may be used to provide environmental flexibility. Facility kitchens should be located near residential buildings to facilitate room delivery. Importantly, outdoor environments for physical activities (e.g., walking routes) should be provided on facility sites to promote residents’ active living. With good natural ventilation, the outdoor environments may be developed into safe common areas with low risk of infection, which are important to institutionalized senior living (Wang, 2020). The rules of social distancing should be followed, and more research is needed to promote residents’ active living during the difficult time of facility lockdown.

### At the Building Level

To restrict entering and exiting, a reception room for visitor screening should be included in building design. Designated areas for visitor’s stay are suggested to reduce unnecessary interactions. Spaces for storing the materials needed for visitor screening, disinfection, and PPE provide convenience and should be included in design.

Natural ventilation helps reduce the concentration of pathogenic virus in the air and has been strongly suggested in the guidelines for infection control. Associated with functional declines, older adults spend a lot of hours inside their rooms, and thus, natural ventilation in their rooms is of great significance to reduce the risk of infection. The availability of indoor natural ventilation is generally influenced by factors associated with site planning and building design, such as site location, building location, building orientation, window location and size. These factors may or may not be modifiable and should be addressed together with passive design strategies in the context of individual facilities. Meanwhile, the quality of window views should be ensured to facilitate residents’ visual access to nature and the world outside, reducing stress and promoting active living (Ulrich, 1984; Wang & Shepley, 2018). In terms of artificial ventilation, air-conditioning systems need to be integrated with zoning and provide an orderly design of air flows by zone to ensure negative air pressures in contaminated zones. Due to the high concentration of airborne virus emitted from contaminated zones, it is recommended to set up relatively high ceilings in these zones and set the return vent near the ceilings and as far away as possible from the supply vent. Guidelines for designing wards for infectious diseases should be used. Window air conditioners may be considered to ensure dedicated air-conditioning.

In a contaminated zone, household waste should be located in a designated place and be separated from the waste from other areas. Routes for waste collection need to be carefully planned at both the site and building levels to prevent virus spread. To develop dedicated circulation systems, behavior maps of the residents, staff, and visitors should be carefully analyzed to identify the overlaps and find the methods for necessary separation. The locations and amount of elevators and staircases need to be appropriately planned.

### At the Room Level

Single rooms are required for patients and suggested for staff’s facility stay. To prevent COVID-19 spread, frequent natural ventilation (30 min per 12 hr) at the room level has been suggested in the guidelines. In case it is inconvenient to conduct natural ventilation through window, mechanical ventilation for outdoor air exchange should be applied and necessary equipment at the room level and spaces for installation were recommended.

Rooms for quarantine should be private and with dedicated toilets and baths. Older adults in senior-living facilities generally need care and those in quarantine may need more care due to infection and associated illnesses. It increases the difficulty of infection prevention and control. Size of the rooms should be relatively larger than typical residential rooms in order to provide spaces for care services inside the rooms. Moreover, spaces for disinfection inside and outside these rooms and related equipment should be provided.

In a resident’s room, access to telemedicine facilitates care, especially for immediate responses to urgent patient safety issues is critical. Internet, television, radio, and reading materials are necessary in order to keep the resident intellectually active and socially supported through tele-interaction with families and friends. Moreover, in dining rooms, necessary engineering separations such as retractable screens may be considered to reduce the risk of droplets and provide the flexibility of space use. A signage system focusing on infection control should be included in design.

## Conclusion

There are practical things we can do to respond to COVID-19 spread, especially for senior-living residents in China, the United States, and all countries. Proper planning and design of the built environment promote strategies for infection control. The guidelines applied in China provide references and the empirical experiences using them have meaningful implications for practice. At the site, building, and room levels, environmental planning and design factors help to prevent and control the virus. Special attention should be given to zoning, natural ventilation, air-conditioning, circulation systems, telemedicine, and tele-interaction. Environmental flexibilities should be considered to balance the changing needs for infection control. These findings can be used to guide the new design, renovation, and modification of senior-living facilities to prevent and control COVID-19 and future public health emergencies.

***Special attention should be given to zoning, natural ventilation, air-conditioning, circulation systems, telemedicine, and tele-interaction***.

Due to limited data availability, the amount and coverage of data collected in this research may not fully represent senior-living professionals’ experience using the guidelines applied in China. However, the need for effective infection control strategies has crossed geographical and national boundaries. The planning and design of environments deserve attention. Through proper planning and design of the built environment, emergency management strategies for infection control used in the pandemic can be gradually transformed into sustainable and effective long-term strategies.

## Implications for Practice

This research developed guidelines for planning and designing environments for infection control in senior-living facilities. For health and care, infection-control factors should be given priority in the design and development of environments for senior living. Due to varied population densities and social economic situations, the buildings and outdoor environments in senior-living facilities vary by location. For instance, senior-living buildings in large cities may have multiple floors whereas those in suburban areas may be single story. Designers should use the guidelines in the context of individual projects and collaborate with senior-living professionals to refine and improve design.

- In site planning, a loading zone or area for material handover outside the facility and a dedicated parking area inside the facility for visiting vehicles should be planned to keep virus from entering the facility.
- The clean (e.g., residential rooms), semi-clean (e.g., medical station), contaminated (patient areas) environmental zones, and dedicated circulation systems for each zone, including the routes for waste collection, should be planned and organized in harmony with the outdoor environments for senior living. Dedicated air-conditioning systems should be developed for each zone to ensure negative air pressures in the contaminated zone, which should be located downwind (in the same direction toward which prevailing winds blow) from other buildings on the site or other spaces in the same building.
- Dedicated areas for staff’s facility stay during lockdown should be planned.
- Accessible outdoor environments should be developed to facilitate residents’ physical activities such as walking on the site.
- Together with passive design strategies, natural ventilation should be promoted for infection control. The factors of building location, building orientation, and window orientation and size should be considered for natural ventilation. Mechanical ventilation directly to the outdoor air for air exchange at the room level should be included in design.
- The amount and locations of elevators, stairs, and corridors should be carefully considered in order to develop dedicated circulation systems for each zone.
- Rooms for quarantine should have a relatively high ceiling and large in size to facilitate safe emission of contaminated air and medical care services. Guidelines for designing wards for infectious diseases may be used.
- Rooms and spaces for internet infrastructure should be included in design to facilitate telemedicine and tele-interaction.
